# Effects of d‐galactose‐induced ageing on the heart and its potential interventions

**DOI:** 10.1111/jcmm.13472

**Published:** 2018-01-24

**Authors:** Cherry Bo‐Htay, Siripong Palee, Nattayaporn Apaijai, Siriporn C. Chattipakorn, Nipon Chattipakorn

**Affiliations:** ^1^ Cardiac Electrophysiology Research Training Center Faculty of Medicine Chiang Mai University Chiang Mai Thailand; ^2^ Department of Physiology Faculty of Medicine Cardiac Electrophysiology Unit Chiang Mai University Chiang Mai Thailand; ^3^ Center of Excellence in Cardiac Electrophysiology Chiang Mai University Chiang Mai Thailand; ^4^ Department of Oral Biology and Diagnostic Science Faculty of Dentistry Chiang Mai University Chiang Mai Thailand

**Keywords:** d‐galactose, heart, ageing, mitochondria

## Abstract

Ageing is a strong independent risk factor for disability, morbidity and mortality. Post‐mitotic cells including those in the heart are a particular risk to age‐related deterioration. As the occurrence of heart disease is increasing rapidly with an ageing population, knowledge regarding the mechanisms of age‐related cardiac susceptibility and possible therapeutic interventions needs to be acquired to prevent advancing levels of heart disease. To understand more about the ageing heart, numerous aged animal models are being used to explore the underlying mechanisms. Due to time‐consuming for investigations involving naturally aged animals, mimetic ageing models are being utilized to assess the related effects of ageing on disease occurrence. d‐galactose is one of the substances used to instigate ageing in various models, and techniques involving this have been widely used since 1991. However, the mechanism through which d‐galactose induces ageing in the heart remains unclear. The aim of this review was to comprehensively summarize the current findings from *in vitro* and *in vivo* studies on the effects of d‐galactose‐induced ageing on the heart, and possible therapeutic interventions against ageing heart models. From this review, we hope to provide invaluable information for future studies and based on the findings from experiments involving animals, we can inform possible therapeutic strategies for the prevention of age‐related heart diseases in clinical settings.

**Table:** 

**•Introduction**
**•Effects of** **d** **‐galactose administration on cardiac senescence markers**
**•Effects of** **d** **‐galactose administration on cardiac oxidative stress and antioxidants**
**•Effects of** **d** **‐galactose administration on cardiac mitochondria**
**•Effects of** **d** **‐galactose administration on cardiac apoptosis**
**•Effects of** **d** **‐galactose administration on intracellular calcium, cardiac proteins, and cardiac function**
**•Effects of therapeutic interventions on the** **d** **‐galactose‐induced ageing heart**
**•The future perspective of ageing heart research**
**•Conclusion**
**•Acknowledgements**
**•Conflict of interests**

## Introduction

The ageing population is growing rapidly across the world, and cardiovascular diseases are the leading cause of death in elderly people worldwide 1. Ageing is described by the progressive loss of physiological functions and is considered as being the chief risk factor for the progress of age‐linked diseases including cardiovascular disease 2. Therefore, understanding the causes and mechanisms associated with age‐related cardiovascular diseases is of considerable importance. Many experimental indications have shown that the characteristics of premature ageing induced by chronic d‐galactose exposure are similar to those in natural ageing in rodents 3, 4, 5. Galactose is a monosaccharide sugar, and although it has two forms, the d and l forms, the body can metabolize only the d form of galactose. There are many d‐galactose‐enriched foods including milk, chocolate, peanut, honey, cheese, yogurt, cherries, kiwi and celery. The recommended daily allowance of the intake of d‐galactose is 50 g per day for a healthy adult, the 50 g being eliminated about 8 hr after ingestion. As long as galactose‐metabolizing enzymes in the body such as galactokinase, galactose 1 uridyltransferase and epimerase are functioning efficiently, the body can effectively metabolize the galactose ingested 6. However, if an abnormal accumulation of d‐galactose occurs in the body as occurs in the injection of d‐galactose‐induced mimetic ageing models, it can cause harmful effects in the body.

The purpose of this review was to comprehensively summarize the studies published on PubMed (https://www.ncbi.nlm.nih.gov/pubmed), under the search terms of ‘d‐galactose and heart and mitochondria’, ‘d‐galactose and heart and ageing’, and ‘d‐galactose and heart’. All these studies have explored the mechanisms of d‐galactose‐induced ageing on the heart and its potential interventions. The common findings as well as the controversial outcome regarding the effects of d‐galactose‐induced ageing on the heart are comprehensively presented and discussed.

## Effects of d‐galactose administration on cardiac senescence markers

Administration of d‐galactose in mice and rats at the age of 2–5 months old, at the dose of 60–150 mg/kg/day, for 6–8 weeks, can increase senescence markers in cardiac tissue 3, 7, 8. For *in vitro* study, 5 g/l of d‐galactose was added to neonatal SD rat cardiomyocytes for 48 hr to induce ageing 9. d‐galactose is a reducing sugar, and when it accumulates in the body, it can react with the free amines of amino acids in proteins and peptides to form a Schiff base, an unstable compound. If this situation continues for subsequent months, the compounds are oxidized and become very stable. These are known as advanced glycation end products (AGEs) 10. AGEs increase during ageing and have been regarded as one of the senescence markers 11, 12. Accumulating evidence proposes that AGEs are interacting with specific receptors (RAGE) in many cell types, including cardiac cells, and induce the activation of a downstream nuclear factor kappa‐B (NF‐κB), and other signalling pathways resulting in generation of reactive oxygen species (ROS), which could accelerate the ageing process 10, 12. In addition, injections of d‐galactose lead to acceleration of an ageing phenotype that is manifested by an increase in senescence‐associated β‐galactosidase (SA β‐gal) staining, SA β‐gal expression and β‐gal‐positive cell expression 8. Furthermore, the mechanisms involved in senescence usually include the p53 or p16 tumour suppressor pathways. Both p53 and p21 expressions were up‐regulated in the d‐galactose‐induced ageing mice model 7. Senescent cells accumulate in the cardiac tissues, leading to reduced regenerative capacity and increased low‐grade inflammation, subsequently inducing ageing and age‐related diseases. Although cellular senescence can be induced by a variety of stress‐induced signalling pathways, oxidative stress caused by exogenous d‐galactose may be the key factor in increasing senescence markers in cardiac tissue 13. The summary of all these findings is shown in Table 1.

**Table 1:** Effects of d‐galactose administration on cardiac senescence markers

Study model	Age	d‐galactose dose (mg/kg/day)	Route	Duration	Major findings	Interpretation	Ref
Wistar rats	5 months	60	IP injection	6 weeks	↑ AGE protein level	d‐galactose increased cardiac senescence.	3
SD rats	2.5 months	150	IP injection	8 weeks	↑ SA‐β‐gal staining ↑ SA‐ β‐gal expression ↑ p21 protein expression	d‐galactose increased cardiac senescence.	8
C57BL/6J mice	2 months	50	SC injection	8 weeks	↔ p16 expression ↑ p53 expression ↑ p21 expression	d‐galactose increased cardiac senescence through p53‐p21 signalling pathway.	7
Wistar rats	5 months *versus* 24 months	60	IP injection	6 weeks	↔ AGE protein level	d‐galactose aged rats shared similarities in senescence protein levels with naturally aged rats.	3
Neonatal SD rats cardiomyocytes	–	5 g/l	–	2 days	↑ β‐gal‐positive cells ↑ AGE content	d‐galactose increased cardiomyocyte senescence.	9

SD rats, Sprague‐Dawley rats; IP, intrapertioneal, SC, subcutaneous; AGEs, advanced glycation endproducts; SA‐β‐gal, senescence‐associated β‐galactosidase; β‐gal, β‐galactosidase.

## Effects of d‐galactose administration on cardiac oxidative stress and antioxidants

**Figure 1:**
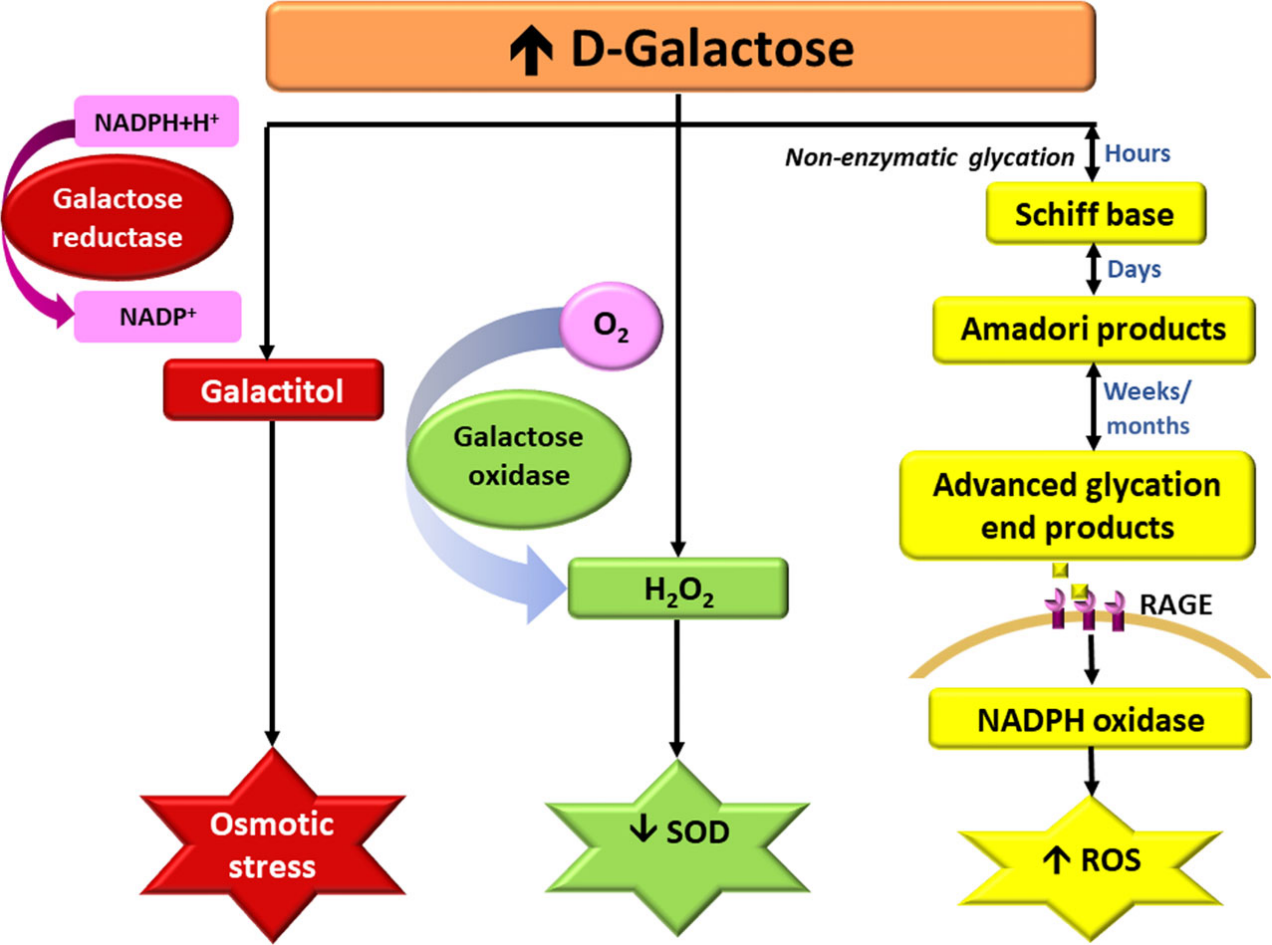
Summary of how d‐galactose induces oxidative stress. Excess d‐galactose is reduced by galactose reductase to form galactitol which can lead to osmotic stress. Additionally, high level of d‐galactose can be oxidized by galactose oxidase to yield hydrogen peroxide; increased hydrogen peroxide causes a decrease in antioxidant enzymes (SOD). Furthermore, d‐galactose can initiate non‐enzymatic glycation reactions to form advanced glycation end products (AGEs) after weeks or months. When AGEs react with their receptors (RAGE), ROS production occurs through NADPH oxidase activation. H_2_O_2_, hydrogen peroxide; SOD, superoxide dismutase; RAGE, receptor for advanced glycation end products; ROS, reactive oxygen species.

Evidence shows that d‐galactose administration increased expression of oxidative stress and decreased expression of antioxidants 3, 7, 8. The doses of d‐galactose involved varied between 50 and 400 mg/kg/day, and duration of the studies was from 6 to 8 weeks 3, 7, 8, 14, 15. Although there are three different metabolic pathways specific to d‐galactose in the body 6, the main pathway is the Leloir pathway. In addition, excess d‐galactose can be converted to galactitol by galactose reductase. Galactitol cannot be further metabolized resulting in increased accumulation in the cells which can affect normal osmotic pressure and cause deterioration of the antioxidant defence system, thus allowing a build‐up of more free radicals 4. In the third pathway, excessive levels of d‐galactose are oxidized by galactose oxidase into reactive aldehydes and hydrogen peroxide. These pathways are illustrated in Fig. 1.

Previous studies have established that oxidative damage by hydrogen peroxide (H_2_O_2_) and a superoxide anion (O_2_^−^), accumulated from excessive d‐galactose metabolism, is a major factor in accelerating mechanisms which contribute to ageing 4, 5. Impaired redox homeostasis due to the increased formation of ROS is widely accepted as a major hallmark of the multifactorial process of ageing 16, 17. Even though ROS have an important role in maintaining normal cell function, excessive ROS produced by d‐galactose metabolism can attack and damage proteins, lipids and DNA, leading to increased protein, lipid and DNA peroxidation 3, 14. Generally, oxidized proteins are degraded by proteolysis, but excess can escape from degradation and form high molecular weight aggregates and accelerate the cardiac ageing process. Major antioxidant systems in cardiac tissue such as total thiol groups and non‐protein thiol groups were significantly reduced, whereas advanced oxidation protein products such as protein carbonyl groups, protein‐bound dityrosine; kynurenine and *N*‐formylkynurenine were increased, indicating that impaired cellular redox homeostasis plays a crucial role in d‐galactose‐induced cardiac ageing 3, 14.

It has also been shown that persistent oxidative stress caused by d‐galactose excess is related to lower ferric reducing antioxidant power and reduced activity of Cu‐Zn superoxide dismutase (Cu‐Zn SOD), enhancing oxidative damage in myocardial tissue (see Fig. 1) 3. In support of this finding, decreased levels of endogenous antioxidant enzymes such as SOD 7, 8, glutathione peroxidase 7, and reduced glutathione levels 14 were demonstrated in d‐galactose‐treated rats and mice. In addition, increased DHE fluorescence staining, decreased endogenous hydrogen sulphide production, endogenous hydrogen sulphide producing enzyme cystathionine γ‐lyase (CSE), and nitric oxide (NO) 7, and decreased haem oxygenase‐1 (HO‐1) 8 were also found in d‐galactose‐induced cardiac ageing models.

Increased lipid peroxidation markers, lipid hydroperoxides (L‐OOH), conjugated dienes (CD) and malondialdehyde (MDA), have been reported in 5‐month‐old Wistar rats after they were given d‐galactose injections for 6 weeks 3. This was also found to be the case in 3‐month‐old Sprague‐Dawley rats 14. Initial lipid peroxidation products L‐OOH and CDs are formed due to reactions between polyunsaturated fatty acids and ROS, and the reactivity of L‐OOH could damage tissue proteins. L‐OOH‐modifying cardiac proteins are assumed to participate in cardiac dysfunction by the alteration of cardiac proteins 18. The final lipid peroxidation product, MDA, can lead to compromisation of the antioxidant defence system 19.

It has been shown that a 60 mg/kg/day dose of d‐galactose administration for 6 weeks induced oxidative DNA damage in cardiac tissue by increasing the formation of 8‐hydroxy‐2′‐deoxyguanosine (8‐OHdG) 3. Guanosine is the most susceptible DNA nucleobase to be oxidized by ROS, and the increase in protein oxidation activity in the d‐galactose‐treated group could cause oxidative damage of this DNA base (guanosine), and be ultimately responsible for the formation of 8‐OHdG in myocardial nuclei. Moreover, oxidative damage of DNA could speed up telomere shortening and accelerate the ageing process 20. In addition, it has been shown that rats undergoing 6 weeks of d‐galactose administration to induce mimetic ageing developed the characteristics of oxidative stress and antioxidants similar to those of 24‐month‐old naturally aged rats, indicating that the d‐galactose‐induced ageing model can be considered as being a reliable experimental model for cardiac senescence 3. The details of all these findings are summarized in Table 2.

**Table 2:** Effects of d‐galactose administration on cardiac oxidative stress and antioxidants

Study model	Age	d‐galactose dose (mg/kg/day)	Route	Duration	Major findings	Interpretation	Ref
Kunming mice	1–1.5 months	125	SC injection	10 weeks	↔ SOD and NO	d‐galactose had no effect on the antioxidants.	15
SD rats	2.5 months	150	IP injection	8 weeks	↓ Antioxidants including HO‐1 and SOD‐1 protein expressions	d‐galactose reduced antioxidants.	8
Wistar rats	5 months	60	IP injection	6 weeks	↑ Protein oxidation markers including AOPP, PCO, DT, KYN and N‐FKYN ↑ Lipid peroxidation markers including L‐OOH, MDA, and CD ↑ Oxidative DNA damage marker 8‐OHdG ↓ Antioxidant levels of Cu–Zn SOD, FRAP and TSH	d‐galactose increased oxidative stress and reduced antioxidants.	3
SD rats	3 months	400	IP injection	6 weeks	↑ Protein oxidation markers including PCO ↑ Lipid peroxidation markers including MDA ↓ Antioxidants including SOD, GSH‐Px, GSH and total antioxidant capacity	d‐galactose increased protein and lipid peroxidation, and reduced antioxidants.	14
Wistar rats	5 months *versus* 24 months	60	IP injection	6 weeks	↔ Protein oxidation markers including AOPP, PCO, DT, KYN, P‐SH and prN‐FKYN ↔ Lipid peroxidation markers including L‐OOH, MDA, and CD ↔ Oxidative DNA damage markers including 8‐OHdG ↔ Antioxidants including Cu‐Zn SOD, FRAP, NP‐SH and T‐SH levels	d‐galactose aged rats shared similarities in oxidative stress and antioxidant status with the naturally aged rats.	3
C57BL/6J mice	2 months	50	SC injection	8 weeks	↑ Oxidative stress marker including DHE ↓ Antioxidants including H_2_S, NO, CSE, SOD and GPx	d‐galactose increased oxidative stress and reduced antioxidants.	7

SD rats, Sprague‐Dawley rats; IP, intraperitoneal; SC, subcutaneous; SOD, superoxide dismutase; NO, nitric oxide; AOPP, advanced oxidation protein products; PCO, protein carbonyl groups; DT, dityrosine; KYN, kynurenine; N‐FKYN, *N*‐formylkynurenine; L‐OOH, lipid hydroperoxides, MDA, malondialdehyde; CD, conjugated dienes; DNA, deoxyribonucleic acid; 8‐OHdG, 8‐hydroxy‐2′‐deoxyguanosine; Cu‐Zn SOD, Cu‐Zn superoxide dismutase; FRAP, ferric reducing antioxidant power; T‐SH, total thiol groups; DHE, dihydroethidium; H_2_S, hydrogen sulphide; CSE, cystathionine γ‐lyase; GPx, glutathione peroxidase.

Despite these reports, there is a study showing that there was no significant difference between the activity of antioxidant enzymes SOD and NO in cardiac tissue 15. In that study, Kunming mice at the age of 4–6 weeks old were used. Compared to this, the initial age of the rats and mice in other studies was 8–20 weeks old 3, 7, 8, 14, 21, 22. Therefore, the differing findings could be explained by the fact that the starting age of experimental animals is the key determinant to create an effective d‐galactose‐induced ageing model. It has been shown consistently that 12‐week and 22‐week‐old animals were more suitable to use to establish a mimetic ageing model indicated by a significant decrease in antioxidant genes 23.

## Effects of d‐galactose administration on cardiac mitochondria

It is well known that mitochondria are not only the targets of ROS, but also the major sites of intracellular ROS production. This ROS‐triggered oxidative damage can cause mitochondrial dysfunction which in turn yields more ROS. This malicious effect of the ROS yielding process and oxidative mitochondrial damage significantly contributes to the ageing process 24. However, there are only two studies which have reported the effects of d‐galactose administration on cardiac mitochondrial energy production 25, and mitochondrial complex 1 activity 26. In these studies, d‐galactose, at a dose of 100 and 125 mg/kg/day, administered for 6 to 8 weeks, had no significant effects on cardiac mitochondrial energy production and mitochondrial complex 1 activity. The limitations of these two studies are that cardiac mitochondrial functions such as mitochondrial ROS production, mitochondrial swelling and mitochondrial membrane potential and mitochondrial morphology are not carried out. Therefore, further studies are needed to clarify the effects of d‐galactose on cardiac mitochondrial function.

## Effects of d‐galactose administration on cardiac apoptosis

There are three studies stating that d‐galactose increased cardiac apoptosis markers 21, 22, 25. The application of the d‐galactose dose varied from 100 to 150 mg/kg/day, the route of administration was either SC or IP injection, and duration was from 6 to 8 weeks. Two major pathways, such as the mitochondria‐initiated intrinsic pathway and the death receptor‐stimulated extrinsic pathway, have been found to be involved in cardiac apoptosis in mammalian cells 27. The extrinsic apoptotic pathway is often triggered by the Fas ligand leading to induction of the formation of death‐inducing signalling complex (DISC). Through the Fas‐associated death domain (FADD), DISC recruits and cleaves pro‐caspase 8 into active caspase 8, which leads to the activation of a key effector of apoptosis, caspase 3 28, 29. It has been shown that rats in a d‐galactose‐treated group had increased cardiac apoptosis *via* an extrinsic pathway 22. Excessive d‐galactose can be converted to advanced glycation end products (AGEs) *via* the Maillard reaction 10. AGEs interact with its receptors, RAGE, which can increase ROS production *via* NADPH oxidase. NADPH oxidase increases p38 MAP kinases, leading to translocation of nuclear transcription factors (NF‐κB) to the nucleus, where they enhance transcription of inflammatory cascades including that for tumour necrosis factor alpha (TNF‐α) 10. Therefore, the existence of increased extrinsic apoptotic markers may be explained by increased inflammation caused by d‐galactose administration.

d‐Galactose also induces cardiac apoptosis *via* the intrinsic pathway. Increased mitochondrial cytochrome *c* release, increased Bax protein expression, decreased Bcl‐2 protein, and reduced Bcl‐2 and Bax ratio are found in the studies displayed in Table 3. Mitochondria are considered as being the cell apoptosis core by liberating apoptogenic molecules such as cytochrome *c* 27. The relative ratio of the Bcl‐2 family members, pro‐apoptotic and anti‐apoptotic proteins, plays a major part in the intrinsic apoptosis pathway. Although the anti‐apoptotic protein Bcl‐2 inhibits mitochondrial cytochrome *c* release, Bax protein makes holes in the outer mitochondrial membrane and as a result, disruption of mitochondrial membrane integrity occurs. This leads to an increase in the level of mitochondrial cytochrome *c* released into the cytosol and activates caspase 3, a crucial mediator in the final apoptosis pathway, instigating DNA fragmentation and apoptosis 30, 31. All these results are summarized in Table 3.

**Table 3:** Effects of d‐galactose administration on cardiac apoptosis

Study model	Age	d‐galactose dose (mg/kg/day)	Route	Duration	Major findings	Interpretation	Ref
SD rats	3 months	125	SC injection	6 weeks	↑ Cytosol Cyt *c* expression ↔ Mito Cyt *c* expression ↓ Bcl‐2 expression ↓ Bcl‐2/Bax ↔ Bax expression	d‐galactose increased apoptosis and reducing anti‐apoptosis	25
SD rats	3 months	100	SC injection	8 weeks	↑ cardiac Cyt *c* ↑ rate of apoptosis ↑ active caspase 3 ↑ Bax expression ↓ Bcl‐2/Bax	d‐galactose increased apoptosis by reducing anti‐apoptotic protein and increasing apoptotic protein.	21
SD rats	2.5 months	150	IP injection	8 weeks	↑ TUNEL‐positive cells ↑ Cytosol Cyt *c* expression ↔ Mito Cyt *c* expression ↑ Fas, FADD, caspase 8 ↓ p‐Akt, Bcl‐2, Bcl‐xL proteins expressions ↑ Cleaved caspase 3 staining	d‐galactose increased apoptosis by increasing apoptosis, and reducing anti‐apoptotic proteins	22

SD, Sprague Dawley rats; SC, subcutaneous; IP, intraperitoneal; Cyt *c*, cytochrome *c*; mito, mitochondria; Fas, tumour necrosis factor receptor; FADD, Fas‐associated death domain; Bcl‐2, B‐cell lymphoma 2; Bax, Bcl‐2‐associated X protein; TUNEL, terminal deoxynucleotidyl transferase of dUTP Nick End Labeling; Akt, protein kinase; Bcl‐xL, B‐cell lymphoma‐extra large.

## Effects of d‐galactose administration on intracellular calcium, cardiac proteins and cardiac function

Previous studies reported that d‐galactose‐induced ageing rats had increased intracellular calcium levels, increased calcium removal time and reduced calcium removal protein expression 9, 21. An effective d‐galactose dose for increase in the intracellular calcium level in 3‐month‐old SD rats was 100 mg/kg/day by SC injection for 8 weeks 21. Removal of calcium from the cytosol may be delayed by a reduced activity in sarco/endoplasmic reticulum calcium adenosine triphosphate (SERCA) or an increase in the activity of phospholamban (PLB) which is a SERCA‐inhibitory protein. Reduced SERCA2a activity and SERCA2a protein expression, together with increased PLB protein expression, can cause intracellular calcium overload, resulting in diastolic dysfunction. This sequence of events has already been described in naturally aged rats 32, 33, 34. A possible explanation for altered calcium homeostasis in d‐galactose‐treated rats and cardiomyocytes may be AGE accumulation, which can cross‐link to intracellular, cardiac sarcoplasmic reticulum (SR) proteins and results in diastolic dysfunction 12. In addition to this, the formation of AGEs has been shown to decrease the amount of serine 16 phosphorylation of PLB while there was no effect on threonine 17 phosphorylation of PLB in an *in vitro* study 9. This suggests that serine phosphorylation of PLB status might be involved in d‐galactose‐induced altered calcium homeostasis as indicated in Table 4.

**Table 4:** Effects of d‐galactose administration on intracellular calcium, cardiac proteins, cardiac function and morphology

Study model	Age	D‐galactose dose (mg/kg/day)	Route	Duration	Major findings	Interpretation	Ref
SD rats	3 months	100	SC injection	8 weeks	↑ [Ca^2+^]_i_	d‐galactose increased intracellular calcium levels.	21
Kunming mice	1–1.5 months	125	SC injection	10 weeks	Mild‐to‐moderate cardiac adipose tissue hyperplasia ↑ Cardiac inflammatory cells	d‐galactose increased cardiac inflammation and adipose tissue hyperplasia.	15
SD rats	2.5 months	150	IP injection	8 weeks	↑ Cardiomyocyte cross‐sectional area ↓ %EF and %FS	d‐galactose‐induced cardiac hypertrophy and LV dysfunction.	22
Wistar rats	5 months	60	IP injection	6 weeks	Vacuolization in cardiomyocyte cytoplasm Disappearance of myofilaments Pale appearance of cytoplasm	d‐galactose‐induced cardiac hypertrophy.	3
SD rats	2.5 months	150	IP injection	8 weeks	↑ p‐ERK1/2, p‐c‐JUN, p‐JNK, and p‐p38 expressions ↑ NFATc3 and p‐GATA4 expressions ↑ p‐MEK5, p‐ERK5 and STAT3 expressions ↑ BNP and MYH‐ 7 expressions ↓ MYH‐6 expression ‐↑ heart weight ‐↑ LV wall thickening	d‐galactose increased cardiac hypertrophic protein expression, leading to cardiac hypertrophy.	8
Neonatal SD rats cardiomyocytes	–	5 g/l	–	2 days	↑ [Ca^2+^]_i_ ↑ Ca^2+^ removal time ↓ SERCA 2a activity ↓ SERCA 2a protein expression ↓ p‐Ser^16^‐PLN protein expression ↔ p‐Thr^17^‐PLN protein expression	d‐galactose reduced calcium removal proteins, leading to increase intracellular calcium level.	9

SD rats, Sprague‐Dawley rats; SC, subcutaneous; IP, intraperitoneal; [Ca^2+^]_i_, intracellular diastolic calcium; EF, ejection fraction; FS, fractional shortening; LV, left ventricle; ERK, extracellular signal‐regulated kinase; NFATc3, nuclear factor of activated T‐cells c3; GATA4, a protein in encoded by GATA4 gene; c‐Jun, a protein encoded by JUN gene; JNK, c‐Jun N‐terminal kinase; MEK, mitogen activated protein kinase; STAT3, signal transducer and activator of transcription 3; BNP, brain natriuretic peptide; MYH, myosin heavy chain; SERCA, sarcoendoplasmic reticulum calcium ATPase; Ser, serine; Thr, threonine; PLN, phospholamban.

Several studies have demonstrated an increase in cardiac hypertrophy, cardiac inflammatory cells and adipose tissue hyperplasia in d‐galactose‐induced cardiac ageing models 3, 8, 15, 22. Doses of d‐galactose, depicted in Table 4, were from 60 to 150 mg/kg/day for a duration of 6 to 10 weeks. As previously mentioned and summarized in Table 2, d‐galactose can induce increased oxidative stress due to abnormal metabolism such as galactose oxidation and generation of AGE that can lead to ROS overproduction in cardiac tissue which might contribute to cardiac hypertrophy. Moreover, AGE–RAGE interactions in cardiac tissue can activate NF‐κB nuclear translocation and increase inflammatory gene transcription, causing inflammation 12.

**Figure 2:**
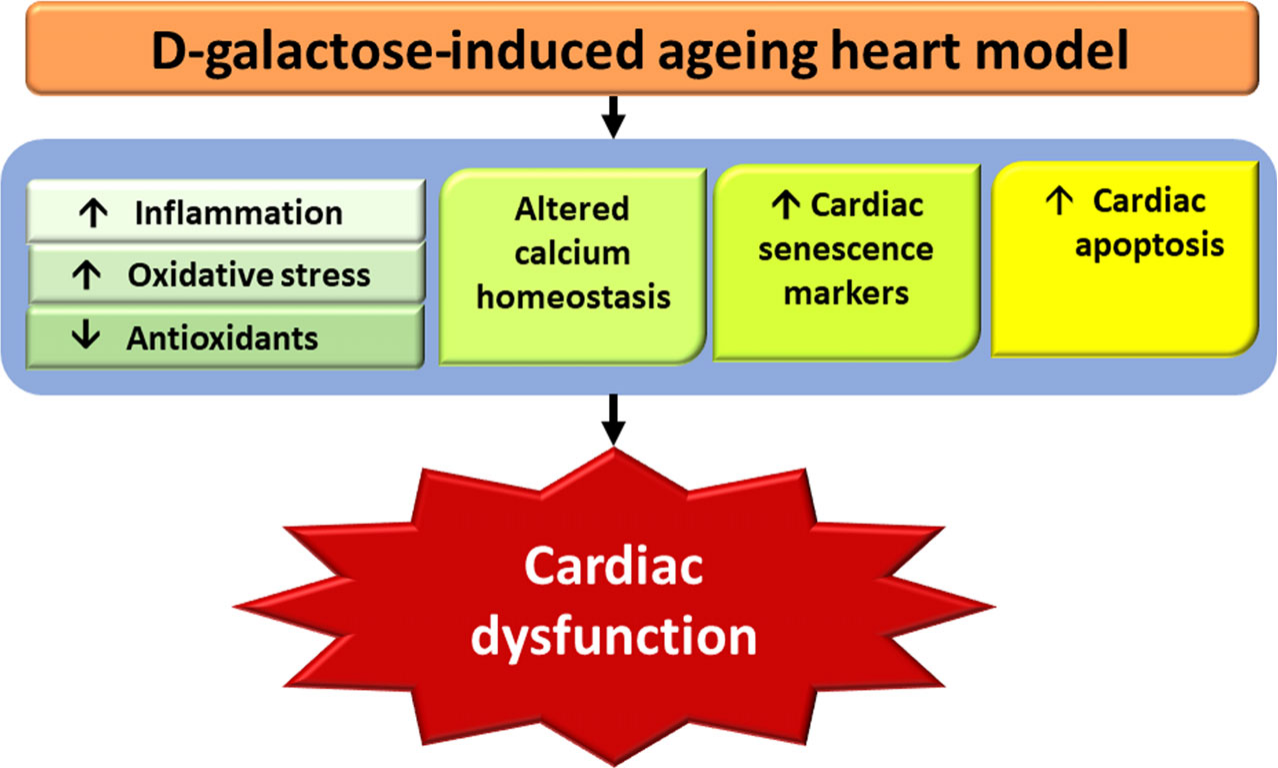
Summary of how d‐galactose induces cardiac dysfunction in ageing heart models. In d‐galactose‐induced ageing heart models, increased cardiac senescence marker expression, increased oxidative stress, decreased antioxidant levels, increased inflammation, increased apoptosis and altered calcium homestasis lead to cardiac dysfunction.

Currently, there is only one study that investigated cardiac function in d‐galactose‐induced aged rats, and the findings showed a decreased left ventricular ejection fraction and fraction shortening in d‐galactose‐induced aged rats when compared to control rats 22. The dose that could induce cardiac dysfunction was 150 mg/kg/day by intraperitoneal injection for 8 weeks as displayed in Table 4. Cardiac dysfunction encountered in d‐galactose‐induced ageing rats may be due to increased oxidative stress, inflammation, apoptosis and altered calcium homeostasis as shown in Fig. 2.

## Effects of therapeutic interventions on the d‐galactose‐induced ageing heart

A variety of therapeutic interventions have been utilized in the d‐galactose‐induced ageing heart in both *in vitro* and *in vivo* studies. These include melatonin hormone, antioxidant nutrients, four Chinese herbal medicines and the hydrogen sulphide donor, sodium hydrosulphide. Of these, the hormone melatonin showed effectiveness in mitochondrial energy production 25. Melatonin is synthesized mainly from tryptophan in the pineal gland. Melatonin is a free radical scavenger and is also regarded as an antioxidative defence against reactive hydroxyl radicals 35, 36. Previous studies have described that melatonin had protective effects in protecting mitochondria in senescence‐accelerated mice (SAM) 37. It was concluded that it could normalize the energy status of heart mitochondria and increase the ATP levels in SAM 38. In addition, an increased Bcl‐2/Bax ratio and reduced cytochrome *c* release were observed following 6 weeks of a 10 mg/kg/day regime of melatonin injections in d‐galactose‐treated rats, indicating that melatonin also had an anti‐apoptotic action 25.

A variety of antioxidants comprising selenium, vitamin E and anthocyanins from purple carrots were tested against oxidative stress induced by d‐galactose 14. The results revealed that both individual treatment and combined treatments led to a decrease in oxidative stress markers, MDA and PCO, and an increase in antioxidant enzymes SOD, glutathione peroxidase and reduced glutathione and a total antioxidant capacity in d‐galactose‐induced aged rats 14. However, combined treatments had greater efficacy in decreasing oxidative stress than those of the individual treatments, likewise a combination of three antioxidants showed greater antioxidant efficacy than a combination of two antioxidants. Selenium is an essential micronutrient, and it is widely accepted that the selenium‐containing enzyme, glutathione peroxidase, is effective against reactive oxygen species 39. Fat‐soluble vitamin E also is regarded as the major antioxidant in the lipid components of cells 40. Anthocyanins showed antioxidant efficacy by donating hydrogen ions to ROS 41 and decreasing lipid peroxidation 42. A combination of those three compounds effected a synergistic action against oxidative stress by anthocyanins activating the glutathione‐related enzymes and increasing the GSH content 43, and selenium boosting GSH which was used in the regeneration of vitamin E 44. It was also reported that their synergism had effects not only by the enzyme systems but also by the non‐enzyme system *via* direct reaction with free radicals and antioxidants 45.

The polysaccharides isolated from Cuscuta chinensis Lam were found to be effective against apoptosis and could reduce the intracellular calcium level in d‐galactose‐induced SD rats 21. In that study, three doses (low, medium and high doses) were used and all of them showed similar efficacy indicated by reduced cytochrome *c* expression, an increased ratio of Bcl‐2 and Bax, increased Bax protein, reduced activity of caspase 3 and a reduced rate of apoptosis. They were also found to decrease calcium accumulation in the cardiac tissue.

The second Chinese traditional herb found in this review was *Dendrobium officinale* (DO). It is regarded as one of the nine precious herbal plant medicines in China. Both a low dose DO (0.32 g/kg, polysaccharide) and a high‐dose DO (1 g/kg, fresh juice) had similar effects in increasing SOD enzymes in d‐galactose‐treated mice 15.

Regarding *Alpinate Oxyphyllae Fructus (AOF)*, two studies indicated that AOF was effective in reducing cardiac hypertrophy and improving cardiac function 8, 22. Of the three doses (low, medium and high), a high dose of AOF (150 mg/kg/day) was more efficacious in improving cardiac function indicated by markedly reduced cardiac senescence, apoptosis and hypertrophic markers, and increasing antioxidants levels in d‐galactose‐treated rats. Of the 80 chemical constituents of AOF, nine secondary metabolites are shown to be concentrated in seeds and fruit capsules. They are flavonoids (*e.g*. tectochrysin, izalpinin, chrysin, apigenin‐4′,7‐dimethyl ether and kaempferide) and sesquiterpenes (*e.g*. nootkatone) and diarylheptanoids (*e.g*. yakuchinone A, yakuchinone B and oxyphyllacinol) 46. As AOF has so many active ingredients, the mechanism of whether the action of each component is receptor mediated or not remained unclear. Further studies are needed to discover whether their actions are mediated through receptors in the body.

The final herbal medicine revealed in this review was Ginkgo biloba extract (EGB761) 9. Although a low dose (5 μg/ml) and medium dose (10 μg/ml) of EGB761 led to a reduction in cardiac senescence, the high dose (20 μg/ml) of EGB761 had a greater efficacy in reducing cardiac senescence and intracellular calcium levels in cultured d‐galactose‐induced ageing rat cardiomyocytes. In that study, the main active compound was 4.2 mg of flavonoid. Many studies have already shown that the flavonoid components have an antioxidant action 47, 48, and the study of 9 proved that a high dose of EGb761 also had an effective action against diastolic dysfunction and had an anti‐ageing action in d‐galactose‐induced accelerated ageing cardiomyocytes.

**Figure 3:**
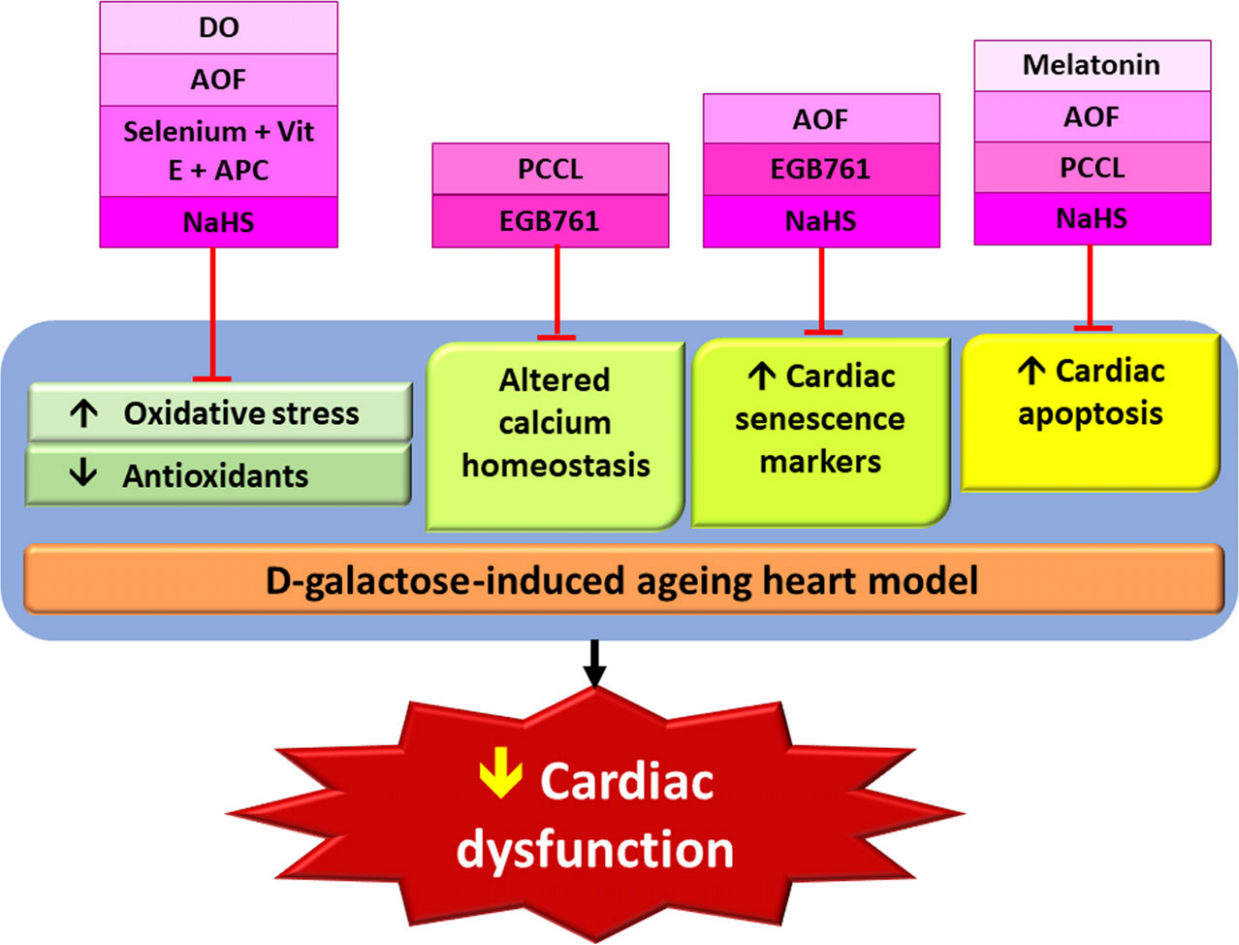
Summary of potential interventions in d‐galactose‐induced ageing heart models. AOF,* Alpinate Oxyphyllae Fructus*; EGB761, Gingko Biloba extract; NaHS, sodium hydrosulphide; DO,* Dendrobium Officinale*; PCCL, polysaccharide isolated from the seeds of Cuscuta chinensis Lam; APC, anthocyanin from purple carrots.

The hydrogen sulphide (H_2_S) donor, sodium hydrosulphide (NaHS), has shown effectiveness in both *in vitro* and *in vivo* studies involving d‐galactose‐induced ageing heart models 7, 49. In the *in vitro* study, 10 g/l of d‐galactose was added to the H9C2 cells for 48 hr to establish an ageing model and subjected to 3‐hr hypoxia/6‐hr reoxygenation. The dose of NaHS was 100 μM and was administered at post‐hypoxia. A post‐conditioning procedure used 5‐min. hypoxia/5‐min. reoxygenation for 30 min. at post‐hypoxia and results were compared between combined the post‐conditioning and NaHS treatment and NaHS treatment alone. The results revealed that combined post‐conditioning and NaHS had a greater efficacy than NaHS alone in protecting aged cardiomyocyte cell death against H/R injury *via* the AMPK‐mTOR autophagic pathway leading to reduced apoptosis 49. Furthermore, NaHS was found to reduce cardiac senescence *via* the p53‐p21 pathway, and in the *in vivo* study, it was not dependent on concentration 7. The summary of all these findings for *in vitro* and *in vivo* studies are included in Tables 5 and 6, respectively. In addition, the mechanisms of how these potential interventions inhibit d‐galactose‐induced cardiac dysfunction are illustrated in Fig. 3.

**Table 5:** Summary of *in vitro* studies on the effects of therapeutic interventions on the d‐galactose‐induced ageing heart

Study model	Age	Intervention	Duration	Major findings					Interpretation	Ref
				Cardiac Senescence	Oxidant/Antioxidant	Apoptosis/Mito/Autophagy	[Ca^2+^]_i_	LV function/remodelling		
Neonatal SD rats cardiomyocytes treated with d‐gal (5 g/l)	–	Ginkgo biloba extract (EGB761) Low dose: 5 μg/ml Medium dose: 10 μg/ml High dose: 20 μg/ml Coincubation	48 hr	Low and medium dose: ↓ β‐gal‐positive cells ↓ AGE Content	–	–	–	–	EGB761 reduced cardiac senescence in a dose‐dependent manner.	9
				*High dose:* ↓↓ β‐gal‐positive cells ↓↓ AGE content			High dose: ↓ [Ca^2^]_i_ ↓ Ca^2+^ removal time ↑SERCA 2a activity and protein ↑ pSer16 PLN protein ↔pThr17 PLN protein			
d‐gal (10 g/l)‐induced aged H9C2 cells, and subjected to 3‐hr hypoxia/6‐hr reoxygenation	–	NaHS (100 μM) was administered at post‐hypoxia Post‐conditioning using 5‐min. hypoxia/5‐min. reoxygenation was carried out for 30 min. at post‐hypoxia Combined post‐conditioning and NaHS at post‐hypoxia	48 hr	–	–	NaHS: ↑ cell viability↓% apoptotic cells ↓ active caspase 3,9 ↓ Cyt *c* ↑ Bcl‐2 ↑ p‐AMPK ↓ p‐mTOR ↑ autophagic vesicles, Beclin‐1, LC3II, Atg5 ↓ p62	–	–	Combined post‐conditioning and NaHS had a greater efficacy than NaHS alone in protecting aged cardiomyocytes against H/R injury *via* AMPK‐mTOR autophagic pathway, leading to reduce apoptosis	49
						Post‐conditioning ↔ cell viability ↔ % apoptotic cells ↔ active caspase 3, 9 ↔ Cyt *c* ↔ Bcl‐2 ↔ p‐AMPK ↔ p‐mTOR ↔ autophagic vesicles, Beclin‐1LC3II, Atg5, p62				
						Combined post‐conditioning and NaHS: ↑↑ cell viability ↓↓ % apoptotic cells ↓↓ active caspase 3,9 ↓↓ Cyt *c* ↑↑ Bcl‐2 ↑↑p‐AMPK ↓↓p‐mTOR ↑↑ autophagic vesicles, Beclin‐1, LC3II, Atg5 ‐↓↓ p62				

D‐gal, D‐galactose; EGB761, Ginkgo Biloba Extract; β‐gal, Beta galactosidase; AGE, advanced glycation end products; [Ca2]_i_, intracellular diastolic calcium; SERCA, sarcoendoplasmic reticulum calcium ATPase; PLN, phospholamban; Ser, serine; Thr, threonine; NaHS, sodium hydrosulphide; Cyt *c*, cytochrome *c*; Bcl‐2, B‐cell lymphoma 2; AMPK, adenosine 5′‐monophosphate (AMP)‐activated protein kinase; H/R, hypoxia/reoxygenation; mTOR, mechanistic target of rapamycin; LC3II, microtubule‐associated protein light chain 3II, Atg5, autophagy protein.

**Table 6:** Summary of *in vivo* studies on the effects of therapeutic interventions on the d‐galactose‐induced ageing heart

Study model	Age	Intervention	Duration	Major findings					Interpretation	Ref
				Cardiac senescence	Oxidant/antioxidant	Apoptosis/mito/autophagy	[Ca^2+^]_i_	LV function/remodelling		
SD rats treated with d‐gal (125 mg/kg)	3 months	Melatonin 10 mg/kg/day IP injection	6 weeks	–	–	↓ cytosol/mito Cyt *C* ↔ Bax expression ↑ Bcl‐2 expression ↑ ATP level	–	–	Melatonin increased ATP levels, reduced apoptosis and increased anti‐apoptotic proteins in d‐galactose‐treated rats.	25
SD rats treated with d‐gal (100 mg/kg)	3 months	PCCL Low dose: 100 mg/kg/day Medium dose: 200 mg/kg/day High dose: 400 mg/kg/day Oral gavage	8 weeks	–	–	↓ Cyt *c* expression ↓ Bax expression ↑ ratio of Bcl‐2/Bax ↓ caspase 3 activity ↓ rate of apoptosis	↓ [Ca^2+^]_i_	–	All doses of PCCL shared similar efficacy in reducing intracellular calcium and apoptosis, and increased anti‐apoptotic protein in d‐galactose‐treated rats.	21
Kunming mice	1–1.5 months	*Dendrobium Officinale* (DO) Low dose: 0.32 g/kg/day High dose: 1 g/kg/day Oral gavage	9 weeks	–	↑ SOD ↔ NO	–	–	–	Both doses of DO shared similar efficacy in increasing antioxidants in d‐galactose‐treated mice.	15
SD rats treated with d‐gal (150 mg/kg)	2.5 months	*Alpinate Oxyphyllae Fructus* (AOF) Low dose: 50 mg/kg/day Medium dose: 100 mg/kg/day High dose: 150 mg/kg/day Oral gavage	10 weeks	*Low‐dose AOF:* ↔ SA‐β‐gal staining ↓ p21 expression	*Low‐dose AOF:* ↔ HO‐1 and SOD	*Low‐dose AOF:* ↑ mito Cyt *c* ↔ cytosol Cyt *c* ↓ TUNEL‐positive cells ↔ Bax ↑ p‐Akt ↓ cleaved caspase 3 staining	**–**	*Low‐dose AOF:* ↓ heart weight ↓ cardiomyocyte cross‐sectional area ↑ %EF and %FS	AOF dose‐dependently improved cardiac function *via* reduced cardiac senescence, apoptosis, hypertrophic markers, and increased antioxidants levels in d‐galactose‐treated rats.	8, 22
				*Medium dose AOF:* ↔ SA‐β‐gal staining ↓ p21 expression	*Medium dose AOF:* ↑ HO‐1 and SOD	*Medium dose AOF:* ↑ mito Cyt *c* ↓ cytosol Cyt *c* ↓ TUNEL‐positive cells ↔ Bax ↑ p‐Akt ↓ caspase 8 ↓ cleaved caspase 3 staining		*Medium dose AOF:* ↓ heart weight ↓ LV wall thickness ↓ cardiomyocyte cross‐sectional area ↑ %EF and %FS		
				High‐dose AOF: ↓ SA‐β‐gal staining ↓ p21 expression	High‐dose AOF: ↑↑ HO‐1 and SOD	High‐dose AOF: ↑ mito Cyt *c* ↓ cytosol Cyt *c* ↓ TUNEL‐positive cells ↓ Bax ↑ p‐Akt ↓↓ Fas and caspase 8 ↓↓ cleaved caspase 3 staining		High‐dose AOF: ↓ heart weight ↓ LV wall thickness ↓ cardiomyocyte cross‐sectional area ↑↑ %EF and %FS		
SD rats d‐gal (400 mg/kg)	3 months	SeMSC: 4.5 μg/kg/day Na_2_SeO_3_: 4.5 μg/kg/day SeY: 4.5 μg/kg/day Vit E (α‐tocopherol acetate): 8.4 mg/kg/day Anthocyanin: 100 mg/kg/day oral gavage	6 weeks	–	*All intervention groups:* ↓ MDA ↓ PCO ↑ SOD ↑ GSH‐Px ↑ GSH ↑ Total antioxidant capacity	–	–	–	Combination of Selenium compound, vitamin E, and anthocyanin showed better efficacy in decreasing oxidative stress and increasing antioxidants than individual treatment alone or combination of two antioxidants in d‐galactose‐treated rats.	14
d‐gal + Vit E + APC *versus * d‐gal + SeMSC/d‐gal + Na_2_SeO_3_/d‐gal + SeY/d‐gal + Vit E/d‐gal + APC					*d* *‐gal + Vit E + APC:* ↓ MDA ↔ PCO ↔ SOD ↔ GSH‐Px ↑ GSH ↔ Total antioxidant capacity					
d‐gal + SeMSC + Vit E + APC/d‐gal + Na_2_SeO_3_ + APC + Vit E/D‐gal +SeY + Vit E + APC *versus* D‐gal + Vit E + APC					Combination of selenium, Vit E and anthocyanin groups had: ↓ MDA ↔ PCO ↑ SOD ↑ GSH‐Px ↑ GSH ↑ Total antioxidant capacity					
D‐gal + SeMSC + Vit E + APC/D‐gal + SeY + Vit E + APC *versus* D‐gal + Na_2_SeO_3_+ APC + Vit E					Organic selenium (SeMSC or SeY) groups had: ↔ MDA ↔ PCO ↔ SOD ↑ GSH‐Px ↔ GSH ↔ Total antioxidant capacity					
C57BL/6 mice treated with d‐gal (50 mg/kg)	2 months	NaHS Low dose: 10 μmol/l Medium dose: 50 μmol/l High dose: 100 μmol/l IP injection	8 weeks	*Low‐dose NaHS:* ↔ p16 ↓ p53 ↓ p21	*Low‐dose NaHS:* ↔ H_2_S, CSE, CBS, and 3‐MST	–	–	–	NaHS reduced cardiac senescence *via* p53‐p21 pathway.	7
				*Medium‐dose NaHS:* ↔ p16 ↔ p53 ↓ p21	*Medium‐ dose NaHS:* ↔ H_2_S, 3‐MST ↑ CSE and CBS					
				*High‐dose NaHS:* ↔ p16 ↓ p53 ↔ p21	*High‐dose NaHS:* ↑ H_2_S, CSE, CBS ↔ 3‐MST					

SD, Sprague‐Dawley rats; d‐gal, d‐galactose; PCCL, polysaccharide isolated from the seeds of Cuscuta chinensis Lam; Cyt *c*, cytochrome *c*; mito, mitochondria; ATP, adenosine triphosphate; Bax, Bcl‐2‐associated X protein; Bcl‐2, B‐cell lymphoma 22; [Ca^2+^]_i_, intracellular diastolic calcium; DO, *Dendrobium Officinale*; SOD, superoxide dismutase; NO, nitric oxide; AOF, *Alpinate Oxyphyllae Fructus*; SA‐β‐gal, senescence‐associated β‐galactosidase; HO‐1, haem oxygenase‐1; TUNEL, terminal deoxynucleotidyl transferase of dUTP nick end labeling; Akt, protein kinase B; EF, ejection fraction; FS, fractional shortening; SeMSC, selenium methylselenocysteine; Se, selenium; SeY, selenium‐enriched yeast; Na_2_SeO_3_, sodium selenite; Vit E, vitamin E; APC, anthocyanin extracts from purple carrot; MDA, malondialdehyde; PCO, protein carbonyl groups; GSH‐Px, glutathione peroxidase; GSH, reduced glutathione; NaHS, sodium hydrosulphide; H_2_S, hydrogen sulfide; CSE, cystathionine γ‐lyase; CBS, cystathionine β‐synthase; 3‐MST, 3‐mercaptopyruvate sulphur transferase.

## The future perspective of ageing heart research

The prevalence of obesity continues to increase worldwide even with significant efforts to reduce the rates of obesity. Obesity is also one of the major risk factors for cardiovascular diseases. As the number of the obese elderly people is increasing, it will be more useful in the clinical setting if the future research will target on effects of ageing and obesity fostered cardiac dysfunction. To be able to develop therapies aiming to reduce ageing and obesity‐related cardiovascular diseases, more mechanistic insights of how ageing and obesity induced mitochondrial dysfunction by assessing cardiac mitochondrial ROS production, mitochondrial membrane potential changes and mitochondrial swelling and mitochondrial dynamics changes by determination of mitochondrial fusion and fission proteins will still need to be explored in ageing obese animals. In addition to focusing on cardiac mitochondria, investigation of oxidative stress marker, inflammatory marker and apoptosis marker expressions will give a better picture for how ageing and obesity deteriorate heart function in ageing obese animals. Furthermore, ageing is associated with decreased function of autophagy and progressive accumulation of damaged proteins and undigested materials, leading to heart failure in elderly subjects. Therefore, assessment of the role of the autophagy in ageing obese animals may also provide effective therapeutic approaches for future clinical applications. All of these may be accomplished at the pre‐clinical state using the d‐galactose‐induced ageing animal model and improved understanding on the ageing process can be further applied to a clinical setting in the future.

## Conclusion

All the experiments summarized in this review have been carried out *in vivo* or *in vitro,* and based on the evidence accumulated here, d‐galactose treatment can successfully induce cardiac senescence models as indicated by increasing expression of senescence markers 3, 7, 8, 9. The mechanisms of how d‐galactose induced an ageing heart can be identified, according to this review, include those resulting in increased oxidative stress and reduced levels of antioxidants 3, 7, 8, 14, increased apoptosis 21, 22, 25, an alteration in calcium homeostasis 9, 21 and impaired cardiac morphology 3, 8, 15, 22. These mechanisms, separately or severally, lead ultimately to cardiac dysfunction 22. These pathways may be targets for developing strategies for approaching the issues associated with anti‐ageing in the future.

## Conflict of interests

The authors declare no conflict of interest.

## References

[1] Sacco RL , Roth GA , Reddy KS , *et al* The Heart of 25 by 25: achieving the goal of reducing global and regional premature deaths from cardiovascular diseases and stroke: a modeling study from the American Heart Association and World Heart Federation. Circulation. 2016; 133: e674–90. 27162236 10.1161/CIR.0000000000000395

[2] Niccoli T , Partridge L . Ageing as a risk factor for disease. Curr Biol. 2012; 22: R741–52. 22975005 10.1016/j.cub.2012.07.024

[3] Cebe T , Yanar K , Atukeren P , *et al* A comprehensive study of myocardial redox homeostasis in naturally and mimetically aged rats. Age. 2014; 36: 9728. 25384832 10.1007/s11357-014-9728-y PMC4226800

[4] Yanar K , Aydin S , Cakatay U , *et al* Protein and DNA oxidation in different anatomic regions of rat brain in a mimetic ageing model. Basic Clin Pharmacol Toxicol. 2011; 109: 423–33. 21733122 10.1111/j.1742-7843.2011.00756.x

[5] Aydin S , Yanar K , Atukeren P , *et al* Comparison of oxidative stress biomarkers in renal tissues of D‐galactose induced, naturally aged and young rats. Biogerontology. 2012; 13: 251–60. 22179795 10.1007/s10522-011-9370-3

[6] Bosch AM . Classical galactosaemia revisited. J Inherit Metab Dis. 2006; 29: 516–25. 16838075 10.1007/s10545-006-0382-0

[7] Wu W , Hou CL , Mu XP , *et al* H2S Donor NaHS changes the production of endogenous H2S and NO in D‐galactose‐induced accelerated ageing. Oxid Med Cell Longev. 2017; 2017: 5707830. 28512525 10.1155/2017/5707830 PMC5420433

[8] Chang YM , Chang HH , Lin HJ , *et al* Inhibition of cardiac hypertrophy effects in D‐galactose‐induced senescent hearts by alpinate oxyphyllae fructus treatment. Evid Based Complement Alternat Med. 2017; 2017: 2624384. 28479925 10.1155/2017/2624384 PMC5396449

[9] Liu J , Wang J , Chen X , *et al* Ginkgo biloba extract EGB761 protects against aging‐associated diastolic dysfunction in cardiomyocytes of D‐galactose‐induced aging rat. Oxid Med Cell Longev. 2012; 2012: 418748. 22693651 10.1155/2012/418748 PMC3368694

[10] Hegab Z , Gibbons S , Neyses L , *et al* Role of advanced glycation end products in cardiovascular disease. World J Cardiol. 2012; 4: 90–102. 22558488 10.4330/wjc.v4.i4.90 PMC3342583

[11] Song X , Bao M , Li D , *et al* Advanced glycation in D‐galactose induced mouse aging model. Mech Ageing Dev. 1999; 108: 239–51. 10405984 10.1016/s0047-6374(99)00022-6

[12] Frimat M , Daroux M , Litke R , *et al* Kidney, heart and brain: three organs targeted by ageing and glycation. Clin Sci (Lond). 2017; 131: 1069–92. 28515343 10.1042/CS20160823

[13] Sikora E , Arendt T , Bennett M , *et al* Impact of cellular senescence signature on ageing research. Ageing Res Rev. 2011; 10: 146–52. 20946972 10.1016/j.arr.2010.10.002

[14] Li X , Zhang Y , Yuan Y , *et al* Protective effects of selenium, vitamin E, and purple carrot anthocyanins on D‐galactose‐induced oxidative damage in blood, liver, heart and kidney rats. Biol Trace Elem Res. 2016; 173: 433–42. 27025718 10.1007/s12011-016-0681-8

[15] Liang CY , Liang YM , Liu HZ , *et al* Effect of *Dendrobium officinale* on D‐galactose‐induced aging mice. Chin J Integr Med. 2017; 23: 1–9. 10.1007/s11655-016-2631-x 28083812

[16] Uzun D , Korkmaz GG , Sitar ME , *et al* Oxidative damage parameters in renal tissues of aged and young rats based on gender. Clin Interv Aging. 2013; 8: 809–15. 23847413 10.2147/CIA.S46188 PMC3700783

[17] Jin K . Modern biological theories of aging. Aging Dis. 2010; 1: 72–4. 21132086 PMC2995895

[18] Eaton P , Hearse DJ , Shattock MJ . Lipid hydroperoxide modification of proteins during myocardial ischaemia. Cardiovasc Res. 2001; 51: 294–303. 11470469 10.1016/s0008-6363(01)00303-0

[19] Baraibar MA , Liu L , Ahmed EK , *et al* Protein oxidative damage at the crossroads of cellular senescence, aging, and age‐related diseases. Oxid Med Cell Longev. 2012; 2012: 919832. 23125894 10.1155/2012/919832 PMC3483731

[20] Tzanetakou IP , Nzietchueng R , Perrea DN , *et al* Telomeres and their role in aging and longevity. Curr Vasc Pharmacol. 2014; 12: 726–34. 24350925 10.2174/1570161111666131219112946

[21] Sun SL , Guo L , Ren YC , *et al* Anti‐apoptosis effect of polysaccharide isolated from the seeds of Cuscuta chinensis Lam on cardiomyocytes in aging rats. Mol Biol Rep. 2014; 41: 6117–24. 24972571 10.1007/s11033-014-3490-1

[22] Chang YM , Chang HH , Kuo WW , *et al* Anti‐apoptotic and pro‐survival effect of alpinate oxyphyllae fructus (AOF) in a d‐galactose‐induced aging heart. Int J Mol Sci. 2016; 17: 466. 27043531 10.3390/ijms17040466 PMC4848922

[23] Xu Y , Wu T , Jin Y , *et al* Effects of age and jet lag on D‐galactose induced aging process. Biogerontology. 2009; 10: 153–61. 18622715 10.1007/s10522-008-9158-2

[24] Cui H , Kong Y , Zhang H . Oxidative stress, mitochondrial dysfunction, and aging. J Signal Transduct. 2012; 2012: 646354. 21977319 10.1155/2012/646354 PMC3184498

[25] Guo XH , Li YH , Zhao YS , *et al* Antiaging effects of melatonin on the myocardial mitochondria of rats and associated mechanisms. Mol Med Rep. 2017; 15: 403–10. 27959405 10.3892/mmr.2016.6002

[26] Chang L , Liu X , Liu J , *et al* D‐galactose induces a mitochondrial complex I deficiency in mouse skeletal muscle: potential benefits of nutrient combination in ameliorating muscle impairment. J Med Food. 2014; 17: 357–64. 24476218 10.1089/jmf.2013.2830 PMC3961779

[27] Wang C , Youle RJ . The role of mitochondria in apoptosis*. Annu Rev Genet. 2009; 43: 95–118. 19659442 10.1146/annurev-genet-102108-134850 PMC4762029

[28] Liou CM , Tsai SC , Kuo CH , *et al* Cardiac Fas‐dependent and mitochondria‐dependent apoptosis after chronic cocaine abuse. Int J Mol Sci. 2014; 15: 5988–6001. 24722570 10.3390/ijms15045988 PMC4013609

[29] Bang S , Jeong EJ , Kim IK , *et al* Fas‐ and tumor necrosis factor‐mediated apoptosis uses the same binding surface of FADD to trigger signal transduction. A typical model for convergent signal transduction. J Biol Chem. 2000; 275: 36217–22. 10952991 10.1074/jbc.M006620200

[30] Zamzami N , Kroemer G . The mitochondrion in apoptosis: how Pandora's box opens. Nat Rev Mol Cell Biol. 2001; 2: 67–71. 11413468 10.1038/35048073

[31] Condorelli G , Morisco C , Stassi G , *et al* Increased cardiomyocyte apoptosis and changes in proapoptotic and antiapoptotic genes bax and bcl‐2 during left ventricular adaptations to chronic pressure overload in the rat. Circulation. 1999; 99: 3071–8. 10368127 10.1161/01.cir.99.23.3071

[32] Ren J , Li Q , Wu S , *et al* Cardiac overexpression of antioxidant catalase attenuates aging‐induced cardiomyocyte relaxation dysfunction. Mech Ageing Dev. 2007; 128: 276–85. 17250874 10.1016/j.mad.2006.12.007 PMC1847331

[33] Li Q , Wu S , Li SY , *et al* Cardiac‐specific overexpression of insulin‐like growth factor 1 attenuates aging‐associated cardiac diastolic contractile dysfunction and protein damage. Am J Physiol Heart Circ Physiol. 2007; 292: H1398–403. 17085535 10.1152/ajpheart.01036.2006

[34] Guo KK , Ren J . Cardiac overexpression of alcohol dehydrogenase (ADH) alleviates aging‐associated cardiomyocyte contractile dysfunction: role of intracellular Ca2+ cycling proteins. Aging Cell. 2006; 5: 259–65. 16842498 10.1111/j.1474-9726.2006.00215.x

[35] Barlow‐Walden LR , Reiter RJ , Abe M , *et al* Melatonin stimulates brain glutathione peroxidase activity. Neurochem Int. 1995; 26: 497–502. 7492947 10.1016/0197-0186(94)00154-m

[36] Reiter RJ . Oxygen radical detoxification processes during aging: the functional importance of melatonin. Aging (Milano). 1995; 7: 340–51. 8719600 10.1007/BF03324344

[37] Okatani Y , Wakatsuki A , Reiter RJ , *et al* Melatonin reduces oxidative damage of neural lipids and proteins in senescence‐accelerated mouse. Neurobiol Aging. 2002; 23: 639–44. 12009513 10.1016/s0197-4580(02)00005-2

[38] Rodríguez MI , Carretero M , Escames G , *et al* Chronic melatonin treatment prevents age‐dependent cardiac mitochondrial dysfunction in senescence‐accelerated mice. Free Radic Res. 2009; 41: 15–24. 10.1080/10715760600936359 17164175

[39] Naziroglu M . Role of selenium on calcium signaling and oxidative stress‐induced molecular pathways in epilepsy. Neurochem Res. 2009; 34: 2181–91. 19513830 10.1007/s11064-009-0015-8

[40] Naziroglu M , Karaoglu A , Aksoy AO . Selenium and high dose vitamin E administration protects cisplatin‐induced oxidative damage to renal, liver and lens tissues in rats. Toxicology. 2004; 195: 221–30. 14751677 10.1016/j.tox.2003.10.012

[41] Algarra M , Fernandes A , Mateus N , *et al* Anthocyanin profile and antioxidant capacity of black carrots (*Daucus carota* L. ssp. sativus var. atrorubens Alef.) from Cuevas Bajas, Spain. J Food Compos Anal. 2014; 33: 71–6.

[42] Devi PS , Kumar MS , Das SM . DNA damage protecting activity and free radical scavenging activity of anthocyanins from red Sorghum (*Sorghum bicolor*) Bran. Biotechnol Res Int. 2012; 2012: 258787. 22400119 10.1155/2012/258787 PMC3286891

[43] Shih PH , Yeh CT , Yen GC . Anthocyanins induce the activation of phase II enzymes through the antioxidant response element pathway against oxidative stress‐induced apoptosis. J Agric Food Chem. 2007; 55: 9427–35. 17935293 10.1021/jf071933i

[44] Brenneisen P , Steinbrenner H , Sies H . Selenium, oxidative stress, and health aspects. Mol Aspects Med. 2005; 26: 256–67. 16105679 10.1016/j.mam.2005.07.004

[45] Kasaikina MV , Turanov AA , Avanesov A , *et al* Contrasting roles of dietary selenium and selenoproteins in chemically induced hepatocarcinogenesis. Carcinogenesis. 2013; 34: 1089–95. 23389288 10.1093/carcin/bgt011 PMC3643414

[46] Chen F , Li HL , Tan YF , *et al* Different accumulation profiles of multiple components between pericarp and seed of Alpinia oxyphylla capsular fruit as determined by UFLC‐MS/MS. Molecules. 2014; 19: 4510–23. 24727421 10.3390/molecules19044510 PMC6271690

[47] Mozet C , Martin R , Welt K , *et al* Cardioprotective effect of EGb 761 on myocardial ultrastructure of young and old rat heart and antioxidant status during acute hypoxia. Aging Clin Exp Res. 2009; 21: 14–21. 19225264 10.1007/BF03324893

[48] Schneider R , Welt K , Aust W , *et al* Cardiac ischemia and reperfusion in spontaneously diabetic rats with and without application of EGb 761: II. Interstitium and microvasculature. Histol Histopathol. 2009; 24: 587–98. 19283667 10.14670/HH-24.587

[49] Chen J , Gao J , Sun W , *et al* Involvement of exogenous H2S in recovery of cardioprotection from ischemic post‐conditioning *via* increase of autophagy in the aged hearts. Int J Cardiol. 2016; 220: 681–92. 27393850 10.1016/j.ijcard.2016.06.200

